# The Activity of PHMB and Other Guanidino Containing Compounds against *Acanthamoeba* and Other Ocular Pathogens

**DOI:** 10.3390/microorganisms10071375

**Published:** 2022-07-08

**Authors:** Dharanga Ratnayake, Michael Ansah, Enas Al Ani, Wayne Heaselgrave

**Affiliations:** 1 Department of Biomedical Science, University of Wolverhampton, Wolverhampton WV1 1LY, UK; d.r.ratnayake@wlv.ac.uk (D.R.); m.ansah3@wlv.ac.uk (M.A.); e.al-ani2@wlv.ac.uk (E.A.A.); 2 Research Institute in Healthcare Science, University of Wolverhampton, Wolverhampton WV1 1LY, UK

**Keywords:** *Acanthamoeba*, bacteria, fungi, contact lens, PHMB, PHMG, PAPB, guazatine acetate

## Abstract

In recent years, a rise in the number of contact lens users in the UK and worldwide coincided with an increased incidence of microbial keratitis. The aim of this study was to investigate the antimicrobial activities of polyhexamethylene guanidine (PHMG), polyaminopropyl biguanide (PAPB), and guazatine in comparison to the common contact lens disinfectant constituent, polyhexamethylene biguanide (PHMB). The study investigated these compounds against a broad range of organisms, including *Acanthamoeba castellanii*, *Acanthamoeba polyphaga*, *Staphylococcus aureus*, *Pseudomonas aeruginosa,* and *Candida albicans*. This study demonstrated that PHMG, PAPB, and guazatine are equal in activity to PHMB against *Acanthamoeba* trophozoites and cysts. PHMG and PAPB are also equal in activity to PHMB against *S. aureus* and *P. aeruginosa*, whereas PHMG shows significantly better activity than PHMB against *C. albicans* (*p* < 0.001). To our knowledge, this is the first study to demonstrate the effectiveness of PHMB, PHMG, PAPB, and guazatine against *Acanthamoeba* and other ocular pathogens. As alternatives to PHMB, these compounds warrant further investigation for inclusion in contact lens solutions and for the treatment of keratitis.

## 1. Introduction

Contact lens wear is increasing worldwide, with approximately 4.5 million people in the United Kingdom and over 41 million in the United States [1,2]. The growth in contact lens wear worldwide led to an upsurge in the incidence of eye infections, including those affecting the cornea that lead to microbial keratitis, and it is estimated there are 1.5 to 2 million cases per year globally [3]. The causative organisms of keratitis are varied and include gram positive bacteria, including *Staphylococcus aureus*, gram negative bacteria including *Pseudomonas aeruginosa*, fungal pathogens including *Candida albicans*, and *Fusarium keratoplasticum*, as well as the free-living amoeba, *Acanthamoeba* spp. [4].

Contact lens wear can involve hard lenses, which can be worn over months and years, and are cleaned and disinfected daily. Alternatively, there are soft contact lenses which can be in the form of daily disposable lenses, which are discarded each day, and weekly/monthly lenses which require daily cleaning and disinfection. Patients most at risk of keratitis are weekly/monthly lenses wearers through risk factors including poor hand hygiene before handling lenses, overnight wear, and the variability in efficacy of lens cleaning solution [5]. Contact lens cleaning solutions have to meet international standards for activity against bacteria, fungi (ISO 14729), and *Acanthamoeba* (ISO 19045-1). The majority of multipurpose solutions (MPS) available on the market today are based upon polyhexamethylene biguanide (PHMB), which is a biguanide polymer where each monomer consists of a six-carbon hexamethyl chain terminated with a biguanide group, and demonstrates a broad spectrum of activity against bacteria, fungi, and *Acanthamoeba.*

However, in commercially available MPS, activity is variable even in solutions containing similar formulations of antimicrobial agents [6,7,8,9]. To overcome this issue, there are newer formulations of MPS, including ACUVUE™ RevitaLens (JnJvision, Jacksonville, FL, USA), which combines the biguanide alexidine with the quaternary ammonium compound polyquaternium-1 (PQ-1), and Biotrue^®^ (Bausch & Lomb, Rochester, NY, USA), which combines the polyaminopropyl-biguanide (PAPB) with PQ-1 to increase antimicrobial activity. PAPB is a biguanide polymer that is structurally similar to PHMB and differs only where each monomer consists of a three-carbon propyl chain terminated with a biguanide group. The variable activity of biguanides in MPS solutions demonstrates that there is a need to identify alternative compounds for the disinfection of contact lenses.

Besides its use in commercial MPS, PHMB is also widely used topically in the treatment of *Acanthamoeba* keratitis (AK) [10]. Current first-line medical therapy for AK is unlicensed and usually involves a biguanide, which is either chlorhexidine or PHMB as a monotherapy at 0.02% (*v*/*v*) or in combination with 0.1% (*v*/*v*) propamidine or 0.1% hexamidine (*v*/*v*), all administered as eye drops several times per day [11]. Despite the in vitro sensitivity of *Acanthamoeba* to PHMB, the response to treatment is highly variable and requires a protracted treatment regime, which has an average cure time of 5 months, with individual patients varying from 1 to 26.24 months [10]. This demonstrates that there is an urgent need to identify alternative compounds for the treatment of AK.

The aim of this study is to investigate the use of PHMB alongside PAPB and other guanidino-containing compounds against *Acanthamoeba* and other ocular pathogens to identify compounds that show potential for the treatment of AK and for the inclusion in MPS. Other compounds will include polyhexamethylene guanide (PHMG), which is a polymeric compound similar to PHMB where each monomer consists of a six-carbon hexamethyl chain terminated with a guanidino group. Another compound will be guazatine, which is a secondary amine with two eight-carbon octyl chains each terminated with a guanidino group.

## 2. Materials and Methods

### 2.1. Test Organism Strains and Culture

The *S. aureus* (NCTC 10788) and *P. aeruginosa* (NCTC 12924) strains used in this study were obtained from the National Collection of Type Cultures, Porton Down, UK. Both were cultured using tryptic soy agar (VWR Ltd., Leicestershire, UK) and incubated at 32 °C. The fungal pathogen *C. albicans* (NCPF 3179) was obtained from the National Collection of Pathogen Fungi, Porton Down, UK. This was cultured using sabouraud dextrose agar (VWR Ltd., Leicestershire, UK) and incubated at 32 °C. The *Acanthamoeba* strains, *A. polyphaga* (ATCC 30461) and *A. castellanii* (ATCC 50370), were obtained from the American Type Culture Collection (LGC Standards, Teddington, UK). Trophozoites were maintained in tissue culture flasks in Ac#6 medium at 30 °C in a static incubator, and cysts were produced using Neff’s encystment medium (NEM) in tissue culture flasks at 30 °C in a shaking incubator as previously described [7].

### 2.2. Test Compounds

The compounds in this study were obtained from the following manufacturers: Poly-hexamethylene biguanide HCl and polymethyleneguanidine HCl (Carbosynth Holdings Ltd., Compton, UK), polyaminopropyl biguanide (Biomaterials USA, Richmond, VA, USA), and guazatine acetate (Sigma-Aldrich, Gillingham, UK). Concentrations of 1 mg/mL of PHMB, PHMG, PAPB, and guazatine were solubilised in water and filter sterilised using a 0.2 µm syringe filter.

### 2.3. Minimum Trophozoite Inhibitory Concentration (MTIC) and Minimum Trophozoite Amoebicidal Concentration (MTAC) Assay

The test compounds were added to a 96-well microtitre plate (Helena Biosciences, Gateshead, UK) and a series of two-fold serial dilutions of each of the test compounds were performed using ¼ strength Ringer’s solution (Oxoid, Basingstoke, UK). After adjusting the trophozoite cell count to a concentration of 2 × 10^4^ cells/mL, the trophozoites were then re-suspended in an Ac#6 growth medium and 100 µL of cells were then added to all the wells in the plate, resulting in a final trophozoite concentration of 1 × 10^4^ cells/mL. After adding the cells, the final drug concentrations ranged from 500 μg/mL to 0.49 μg/mL. The plate was then incubated at 32 °C for 24 h. The MTIC/MTAC was then determined by comparing the rate of growth, inhibition, or kill against the control, using an inverted microscope (×200). The MTIC and MTAC are defined as the lowest concentration that gave inhibition of growth and cell lysis, respectively, relative to the control. This is easily seen microscopically, as healthy trophozoites remain attached to the bottom of the wells. The control wells received sterile water in place of test compounds, and any differences relative to the control can be confirmed microscopically through the absence of cell division and the presence of cell lysis. 

### 2.4. Minimum Cysticidal Concentration (MCC) Assay

After adjusting the cyst count to a concentration of 2 × 10^4^ cells/mL in ¼ strength Ringer’s solution, 100 µL of cysts were then added to the wells, resulting in a final cyst concentration of 1 × 10^4^ cells/mL. After adding the cysts, the plate was incubated at 32 °C for 48 h. After 48 h of incubation, the liquid was aspirated from the microtitre plate using a Vacusip (Integra Biosciences, Thatcham, UK), leaving behind the cysts that adhered to the bottom of the wells. Wells were then refilled with ¼ strength Ringer’s solution, which was allowed to stand for 10 min before being aspirated. This step was repeated 3 times to ensure removal of the test compounds. Finally, to each well, an *Escherichia coli* suspension at an OD of 0.1–0.2 at 600 nm was added. The plates were then incubated at 32 °C and observed daily for 7–14 days. The MCC was defined as the lowest concentration that gave no excystation relative to the control. The control wells received sterile water in place of test compounds, and any differences relative to the control can be confirmed microscopically through the absence of excystation and cell division. 

### 2.5. Minimum Inhibitory Concentration (MIC) and Minimum Biocidal Concentration (MBC) Assay

The minimum inhibitory concentration (MIC) assay was performed in a 96-well tissue microtitre plate using a similar method as described above for the *Acanthamoeba* to create the range of concentrations. After adjusting the bacteria and fungi to a concentration of 2 × 10^5^ CFU/mL in tryptic soy broth (TSB), the cell suspension was added to give a final cell concentration of 1 × 10^5^ CFU/mL. The plates were incubated at 32 °C for 24 h. The MIC of each compound was determined visually by identifying the lowest concentration of a drug that caused no visible growth (turbidity), and was confirmed using a plate reader at 600 nm. The MBC was determined by subculturing the contents of the wells on the appropriate agar for each organism. 

### 2.6. Composition of Contact Lens Base Solution (CLB)

A contact lens base was prepared by supplementing ¼ strength Ringer’s solution with 0.30 mM of the surfactant Pluronic F127 (Sigma-Aldrich, Gillingham, UK). Before adding EDTA (0.068 mM), NaH_2_PO_4_ (0.15 mM), and Na_2_HPO_4_ (0.84 mM), the pH was adjusted to 7.2 with 1M NaOH before autoclaving.

### 2.7. Acanthamoeba Time-Kill Assay

The rate of kill for each compound was investigated using the time-kill method as previously described [12]. Briefly, this involved exposing trophozoites and cysts to a concentration of a test compound for up to 24 h, which was formulated in a contact lens base solution. At time intervals of 0, 2, 4, 6, and 24 h, 20 μL aliquots were removed from each centrifuge tube in quadruplet, and serial 10-fold dilutions were performed across the plate. Each well was then seeded with *E. coli* (as described above for MCC assay) and the plates were incubated at 32 °C for 7–14 days. On a daily basis, the plates were examined for encystment or trophozoite growth using an inverted microscopy. The number of viable organisms at each time point was calculated using the Spearman–Karber most probable number method [7,13].

### 2.8. Bacterial and Fungal Time-Kill Assay

The rate of kill for each compound was investigated using the time-kill method as previously described [12]. Briefly, this involved exposing bacteria and fungi to a concentration of a test compound for up to 24 h, which was formulated in a contact lens base solution. At time intervals of 0, 2, 4, 6, and 24 h, 20 μL aliquots were removed from each centrifuge tube and serial 10-fold dilutions were performed in Dulbecco’s phosphate-buffered saline (Oxoid, Basingstoke, UK). Then, using the Miles and Misra technique, each dilution was plated onto an appropriate agar and incubated at 32 °C for 24 h [14].

### 2.9. Analysis of Data

To determine the reduction in viable bacteria, fungi, and *Acanthamoeba*, the decrease in viability was plotted as log viability for each of the time points and the reduction was determined relative to the log viability. Statistical analysis was performed using Instat (GraphPad, La Jolla, CA, USA). The log reduction of the compounds was compared to the control and to each other and then analysed using the Tukey–Kramer multiple comparisons test.

## 3. Results

In this study PHMB, PHMG, PAPB, and guazatine were tested against trophozoites and cysts of *A. castellanii* and *A. polyphaga,* as well as other potential ocular pathogens, including *S. aureus*, *P. aeruginosa* and *C. albicans.* Test compounds were first screened to determine the minimum concentration for which they showed activity against the panel of organisms before the kinetics of the inactivation was determined over 24 h in a time-kill study.

### 3.1. MTIC/MTAC/MCC—Acanthamoeba

The results in Table 1 show the antimicrobial activities of PHMB, PHMG, PAPB, and guazatine against *A. castellanii* and *A. polyphaga* (trophozoites and cysts). All compounds showed good activity against trophozoites and cysts with PAPB and PHMB showing the best activity against trophozoites with an MTIC and MTAC of 3.9 and 7.8 µg/mL, respectively, against both species. Against cysts, all compounds were active in the 7.8–15.6 µg/mL range against both species.

### 3.2. MIC/MBC—Bacteria and Fungi

The results in Table 2 show the antimicrobial activities of PHMB, PHMG, PAPB, and guazatine against *P. aeruginosa*, *S. aureus* and *C. albicans.* With *S. aureus*, PAPB and PHMB were the most active, with an MIC/MBC of 2 µg/mL. With *P. aeruginosa*, PHMG showed the lowest MIC at 3.9 µg/mL and guazatine the lowest MBC at 7.8 µg/mL. Finally, with *C. albicans,* PHMG was the most active, with an MIC/MBC of 0.5 µg/mL.

### 3.3. Time-Kill Assays—Acanthamoeba Trophozoites and Cysts

**Figure 1:**
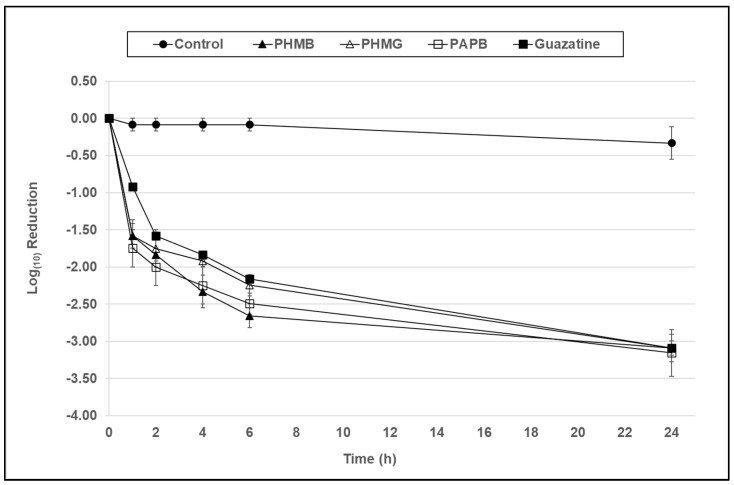
The antimicrobial activity of PHMB, PHMG, PAPB, and guazatine against *A. polyphaga* (ATCC 30461) trophozoites over a 24 h period at a concentration of 7.8 µg/mL formulated in a contact lens base solution. Error bars show the standard error of the mean (SEM) from triplicate experiments.

**Figure 2:**
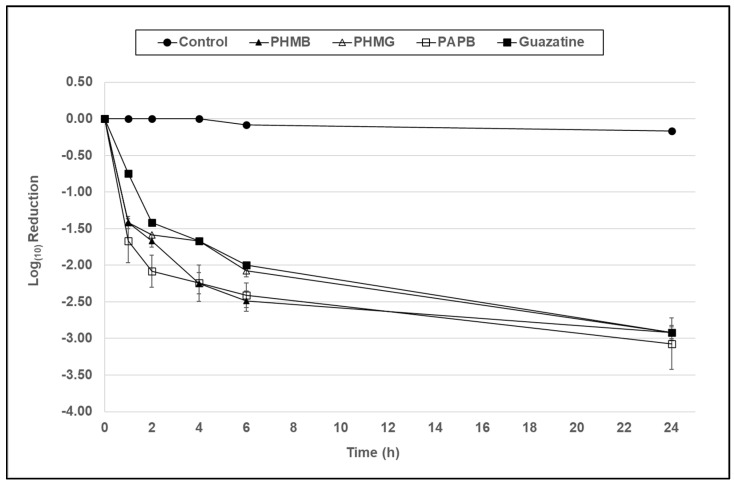
The antimicrobial activity of PHMB, PHMG, PAPB, and guazatine against *A. castellanii* (ATCC 50370) trophozoites over a 24 h period at a concentration of 7.8 µg/mL formulated in a contact lens base solution. Error bars show the standard error of the mean (SEM) from triplicate experiments.

**Figure 3:**
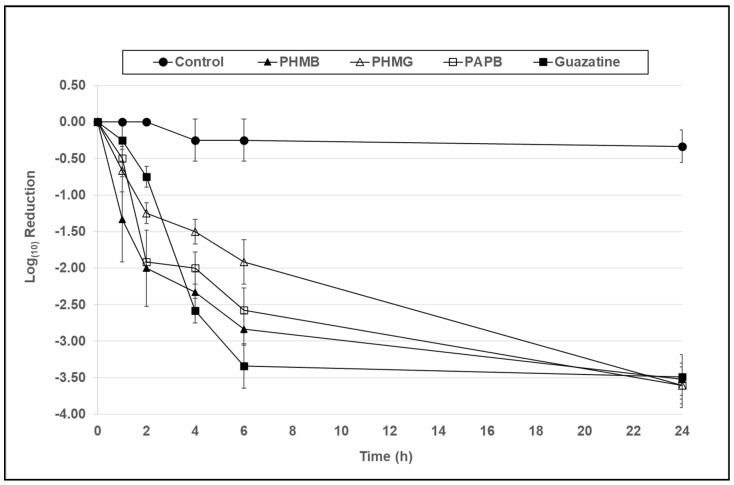
The antimicrobial activity of PHMB, PHMG, PAPB, and guazatine against *A. polyphaga* (ATCC 30461) cysts over a 24 h period at a concentration of 7.8 µg/mL. Error bars show the standard error of the mean (SEM) from triplicate experiments.

**Figure 4:**
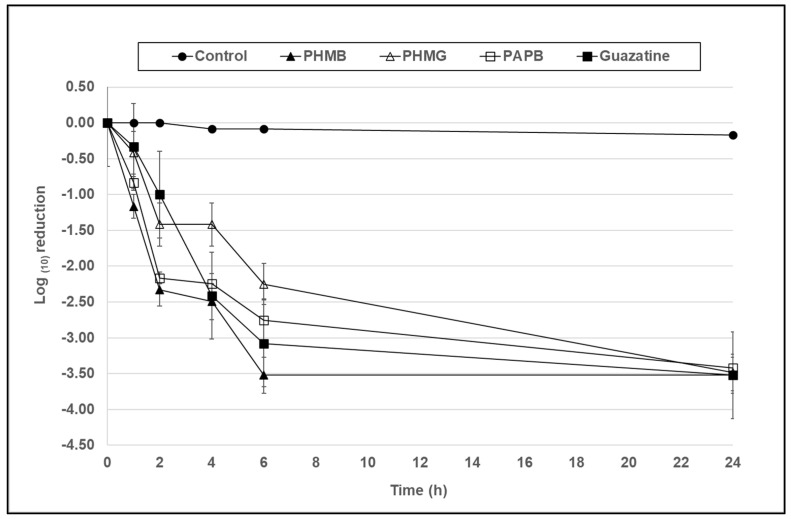
The antimicrobial activity of PHMB, PHMG, PAPB, and guazatine against *A. castellanii* (ATCC 50370) cysts over a 24 h period at a concentration of 7.8 µg/mL. Error bars show the standard error of the mean (SEM) from triplicate experiments.

The antimicrobial activity of PHMB, PHMG, PAPB, and guazatine against cysts and trophozoites of two different *Acanthamoeba* species along with *S. aureus*, *P. aeruginosa*, and *C. albicans* over a 24 h period are displayed in Figure 1, Figure 2, Figure 3 and Figure 4 and Table 3. Based on the results from the screening assays, a concentration of 7.8 µg/mL was selected for the *Acanthamoeba*, and 0.05–0.1 µg/mL for bacterial and fungal assays. The compounds were added to a contact lens base solution containing buffer and surfactant to simulate a contact lens solution. 

In Figure 1 and Figure 2, at a concentration of 7.8 µg/mL, all compounds demonstrated antimicrobial activity against trophozoites of both *Acanthamoeba* species, resulting in a 2–2.6 and 2.9–3.1 log_10_ reduction in viability in comparison to the control at 6 and 24 h, respectively.

In Figure 3, at a concentration of 7.8 µg/mL, PHMB, PHMG, and PAPB demonstrated cysticidal activity against both *Acanthamoeba* species, achieving a 1.9–2.8 and 3.5 log_10_ reduction in viability in comparison to the control at 6 and 24 h, respectively. Guazatine gave the greatest reduction in viability of 3.4 log_10_ at 6 h but was comparable to the rest at 24 h.

In Figure 4, at a concentration of 7.8 µg/mL, PHMG and PAPB demonstrated cysticidal activity against both *Acanthamoeba* species, achieving a 2.2–2.7 and 3.5 log_10_ reduction in viability in comparison to the control at 6 and 24 h, respectively. PHMG and guazatine gave the greatest reduction in viability of 3–3.5 log_10_ at 6 h but were comparable to the rest at 24 h.

### 3.4. Time-Kill Assays—Bacteria and Fungi

In Table 3, at a concentration of 0.05 µg/mL, PHMB, PHMG, and PAPB demonstrated anti-bacterial activity against *S. aureus* in comparison to the control of 4.87–5.61 and 5.61 log_10_ reduction at 6 and 24 h, respectively. Guazatine showed limited activity of 0.5 and 0.72 log_10_ reduction at 6 and 24 h, respectively. 

At a concentration of 0.1 µg/mL, PHMB, PHMG and PAPB demonstrated anti-bacterial activity against *P. aeruginosa* in comparison to the control of 5.63 log_10_ reduction at 6 h, which corresponded to complete inactivation. Guazatine showed limited activity of a 0.2 and 0.23 log_10_ reduction at 6 and 24 h, respectively. 

At a concentration of 0.05 µg/mL, PHMB and PAPB demonstrated anti-fungal activity against *C. albicans* in comparison to the control of 1.41–2.07 and 3.9–4.05 log_10_ reduction at 6 and 24 h, respectively. PHMG gave complete inactivation of 5.16 log_10_ reduction at 6 h. Guazatine showed limited activity of 0.12 and 0.08 log_10_ reduction at 6 and 24 h, respectively.

## 4. Discussion

Keratitis is a potentially sight-threatening infection often seen in contact lens wearers that can be caused by a variety of pathogens, including bacteria, fungi, and free-living amoebae. Weekly and monthly contact lens wearers are required to disinfect their lenses daily, and due to a variety of risk factors, including poor hand hygiene and the choice of MPS, are at risk of infection. AK which is caused by *Acanthamoeba* is of particular concern, as it is able to form a highly resistant cyst that is resistant to disinfection [12], and when a patient contracts AK, it requires a protracted treatment regime with a mean treatment time of many moths to effect a cure [10]. Therefore, there is an urgent need to identify alternative compounds for the inclusion into MPS for contact lens disinfection and for the treatment of AK.

In this study PHMB, PHMG, PAPB, and guazatine were tested against trophozoites and cysts of *A. castellanii* and *A. polyphaga,* as well as other potential ocular pathogens, including *S. aureus*, *P. aeruginosa,* and *C. albicans.* Test compounds were first screened to determine the minimum concentration at which they showed activity against the panel of organisms before the kinetics of the inactivation was determined over 24 h. Based on the results from the screening assay, a concentration of 7.8 µg/mL was selected for *Acanthamoeba* and 0.05–0.01 µg/mL for all other organisms. For the time-kill assays, the compounds were added to a contact lens base solution containing buffer and surfactant to simulate a commercial contact lens solution and to provide enhanced activity.

### 4.1. MTIC/MTAC/MCC—Acanthamoeba

The results in Table 1 show the antimicrobial activities of PHMB, PHMG, PAPB, and guazatine against *A. castellanii* and *A. polyphaga* (trophozoites and cysts). All compounds showed good activity against trophozoites and cysts with PAPB and PHMB, showing the best activity against trophozoites. Against cysts, all compounds were active in the 7.8–15.6 µg/mL range against both species.

The results for PHMB against *Acanthamoeba* observed in this study against cysts are identical to our previously published study, which reported 15.6 µg/mL as the MCC for cysts [10], and similar to this study, which reported 11.87 µg/mL [15]. However, it is higher than that reported in an earlier study, where the authors reported an MCC of 2.38 µg/mL [16]. The reasons for the difference to the 2003 study is likely due to the fact that they used a range of clinical isolates of unknown species, whereas this study used strains of confirmed species taken from a recognised culture collection. 

The results for the MTIC and MTAC of PHMB were between 2 and 3.9 µg/mL, which are similar to our previous study of 1–7.8 µg/mL [10]. The reason for the small difference between the studies is because the incubation period was reduced from 48 h to 24 h in the current study to mirror the time frame used in the time-kill assay. An earlier study from 1994 reported much lower values for MTIC and MTAC of 0.5 and 1.0 µg/mL, respectively [17]. The reason for these lower values is again likely due to the fact that they used a range of clinical isolates of unknown species, whereas this study used strains of confirmed species taken from a recognised culture collection.

To our knowledge, this is the first study to report the minimum activities of PAPB, PHMG, and guazatine against trophozoites and cysts of *Acanthamoeba* spp. Guazatine was previously used as agricultural fungicide and reported to be active against the eukaryotic spore-forming pathogen *Ustilago maydis* in the range of 6.4 µg/mL [18], which is similar to the MTAC observed with *Acanthamoeba* trophozoites in this study. 

### 4.2. MIC/MBC—Bacteria and Fungi

The results in Table 2 show the antimicrobial activities of PHMB, PHMG, PAPB, and guazatine against *P. aeruginosa*, *S. aureus,* and *C. albicans.* With *S. aureus*, PAPB and PHMB were the most active, with an MIC/MBC of 2 µg/mL. With *P. aeruginosa*, PHMG showed the lowest MIC at 3.9 µg/mL, and guazatine the lowest MBC at 7.8 µg/mL. Finally, with *C. albicans,* PHMG was the most active, with an MIC/MBC of 0.5 µg/mL.

PHMB demonstrated a broad range of antimicrobial activity in this study against *S. aureus*, *P. aeruginosa,* and *C. albicans,* achieving MIC values of 2, 7.8, and 7.8 µg/mL, respectively, and MBC values of 2, 15.6, and 15.6 µg/mL, respectively. The values obtained in this study are supported by observations from previous studies where 1 µg/mL was observed against *S. aureus* [19], 7.8–15.6 µg/mL was observed against *P. aeruginosa* [20], and 0.78–1.56 µg/mL was observed against *C. albicans* [21].

Previous MBC studies comparing PHMB and PAPB against *S. aureus* and *P. aeruginosa* were reported [22]. In this previous study, the authors reported an MBC of <50 µg/mL for *S. aureus* and *P. aeruginosa,* but an MBC of 2500 and 10,000 µg/mL for *S. aureus* and *P. aeruginosa,* respectively, for PAPB. The authors stated that they believe that previous studies confused PHMB and PAPB, as they have similar biguanide structures differing only by the methylene spacer. The reason for the lack of activity from the previous study is unknown. However, as part of this study, we purchased PAPB (CAS# 133029-32-0) and PHMB (CAS# 32289-58-0) from manufacturers that supply both compounds (Biomaterials USA, Richmond, VA, USA) and (Carbosynth Holdings Ltd., Compton, UK) to avoid any confusion over the structures. 

Previous MIC studies with PHMG against *C. albicans* were reported as achieving MIC values of 2.5 µg/mL, which is similar to the 0.49 µg/mL reported in this current study [23]. Previous MIC studies with guazatine indicate that guazatine is composed of a mixture of reaction products from polyamines comprising mainly of octa-methylene diamine. The study demonstrated that these different products have varying activity when separated and tested against *C. albicans*. The authors reported that the fractions of the guazatine mixture had an MIC ranging from approximately 7.1 to 28.4 µg/mL, which at the lower end agrees with the 3.9 µg/mL results from this present study [24].

### 4.3. Time-Kill Assays—Acanthamoeba trophozoites and cysts

In Figure 1 and Figure 2, at a concentration of 7.8 µg/mL, all compounds demonstrated significant antimicrobial activity against trophozoites of both *Acanthamoeba* species resulting in a 2–2.6 log_10_ reduction in viability in comparison to the control (*p* < 0.001) at 6 h, which is the standard disinfection time for a contact lens solution. However, there was no significant difference between PHMB, PHMG, PAPB, and guazatine at this 6 h time point (*p* > 0.05).

In Figure 3, at a concentration of 7.8 µg/mL PHMB, PAPB, PHMG, and guazatine demonstrated significant cysticidal activity against *A. polyphaga,* achieving a 1.9–3.4 log_10_ reduction in cyst viability in comparison to the control at the 6 h time point; PHMB, PAPB, and guazatine (*p* < 0.001), and PHMG (*p* < 0.05). Guazatine gave the greatest reduction in viability of 3.4 log_10_ at 6 h, which was significant compared to PHMG (*p* < 0.05), but not compared to PHMB and PAPB (*p* > 0.05).

In Figure 4, at a concentration of 7.8 µg/mL, PHMB, PAPB, PHMG, and guazatine demonstrated significant cysticidal activity against *A. castellanii,* achieving a 2.7–3.5 log_10_ reduction in cyst viability in comparison to the control at the 6 h time point; PHMB, PAPB, and guazatine (*p* < 0.001), and PHMG (*p* < 0.01). PHMB gave the greatest reduction in viability of 3.4 log_10_ at 6 h, however, there was no significant difference to the other compounds at this time point (*p* > 0.05).

To our knowledge, this is the first study to report the activities of PAPB, PHMG, and guazatine against trophozoites and cysts of *Acanthamoeba* spp. and to compare them to PHMB. Previous studies reported the activity of PHMB as part of a time-kill experiment against *Acanthamoeba* cysts, but these are usually run at 1 µg/mL (0.0001% *v*/*v*), as this is the typical concentration of PHMB in a commercial product [7]. In the former study, the authors used exactly the same strains and cyst preparation methods and achieved similar results to this study, achieving 2.80 and 3.50 log_10_ reduction at 6 h for *A. castellanii* and *A. polyphaga,* respectively. In terms of treatment, the cysticidal activity of PHMB in the range of 7.8 µg/mL indicates that the administration of drops at 0.02% (*v*/*v*), which is equivalent to 200 µg/mL, is sufficiently concentrated to have antimicrobial activity in vivo. However, in terms of contact lens disinfection, the cysticidal activity of PHMB in the range of 7.8 µg/mL is below the 1 µg/mL found in many contact lens disinfectants, which indicates that they are unlikely to achieve complete disinfection of cysts. To improve antimicrobial activity of biguanide-based disinfecting solutions, some manufacturers have now added the quaternary ammonium compound polyquaternium-1 (PQ-1) as a second disinfectant.

### 4.4. Time-Kill Assays—Bacteria and Fungi

In Table 3, at a concentration of 0.05 µg/mL, PHMB, PHMG, and PAPB demonstrate significant (*p* < 0.001) anti-bacterial activity against *S. aureus* of 4.87–5.61 log_10_ reduction in viability at 6 h, which is the standard disinfection time for a contact lens solution. Guazatine showed limited activity at 6 and 24 h, and showed no significant difference to the control (*p* > 0.05). There was no significant difference (*p* > 0.05) between PHMB, PHMG, and PAPB at the 6 h time point, but overall, PHMB and PAPB performed slightly better, as they achieved complete inactivation of *S. aureus* at 4 h.

In Table 3, at a concentration of 0.1 µg/mL, PHMB, PHMG, and PAPB demonstrate significant (*p* < 0.001) anti-bacterial activity against *P. aeruginosa* of 5.63 log_10_ reduction in viability at 6 h, which corresponded to complete inactivation. Guazatine showed limited activity at 6 and 24 h, and showed no significant difference to the control (*p* > 0.05). There was no significant difference (*p* > 0.05) between PHMB, PHMG, and PAPB at the 6 h time point, and overall, all performed to a similar level, achieving complete inactivation of *P. aeruginosa* at 2 h. 

In Table 3, at a concentration of 0.05 µg/mL, PHMB and PAPB demonstrate significant anti-fungal activity against *C. albicans* in comparison to the control of 1.41–2.07 log_10_ reduction at 6 h compared to 5.16 log_10_ reduction with PHMG. Guazatine showed limited activity at 6 and 24 h, and showed no significant difference to the control (*p* > 0.05). PHMG demonstrated significantly better activity than PHMB and PAPB within 6 h (*p* < 0.001). There is no significant difference between the activities of PHMB and PAPB against *C. albicans* at 6 h.

Previous time-kill studies with PHMB against *S. aureus*, *P. aeruginosa,* and *C. albicans* using the standardised contact lens disinfection method used in this study (ISO 14729) demonstrated 5, 5, and 3 log_10_ reduction, respectively, for each organism after 1 h [25]. This is faster than the kill observed in this present study, however, the authors were using 1 µg/mL PHMB for all organisms, whereas this current study used 0.05 µg/mL for *S. aureus* and *C. albicans* and 0.1 µg/mL *P. aeruginosa*.

## 5. Conclusions

In conclusion, this study has demonstrated that PAPB, PHMG, and guazatine have similar levels of activity to PHMB against cysts and trophozoites of *A. castellanii and A. polyphaga*. When tested against other ocular pathogens including *P. aeruginosa*, *S. aureus,* and *C. albicans,* guazatine demonstrates limited activity, but PAPB and PHMG show strong activity similar to that of PHMB. Taken together, these observations indicate that further work is needed to evaluate these compounds as potential disinfectants for inclusion in contact lens solutions and their potential for the treatment of AK.

## Figures and Tables

**Table 1:** The minimum trophozoite inhibitory concentration (MTIC), minimum trophozoite amoebicidal concentration (MTAC), and minimum cysticidal concentration (MCC) of PHMB, PHMG, PAPB, and guazatine against *A. castellani* and *A. polyphaga*.

In Vitro Drug Sensitivities (µg/mL)						
Drug	*A. castellanii*			*A. polyphaga*		
	MTIC	MTAC	MCC	MTIC	MTAC	MCC
PHMB	3.9	7.8	15.6	7.8	7.8	7.8
PHMG	7.8	15.6	15.6	3.9	7.8	7.8
PAPB	3.9	7.8	7.8	2.0	3.9	7.8
Guazatine	7.8	15.6	15.6	3.9	7.8	7.8

**Table 2:** The Minimum Inhibitory Concentration (MIC) and Minimum Biocidal Concentration (MBC) of PHMB, PHMG, PAPB and guazatine against *P. aeruginosa*, *S. aureus* and *C. albicans*.

In Vitro Drug Sensitivities (µg/mL)						
Drug	*S. aureus*		*P. aeruginosa*		*C. albicans*	
	MIC	MBC	MIC	MBC	MIC	MBC
PHMB	2.0	7.8	7.8	15.6	3.9	15.6
PHMG	3.9	7.8	3.9	15.6	0.5	0.5
PAPB	2.0	2.0	7.8	15.6	7.8	15.6
Guazatine	2.0	2.0	7.8	7.8	3.9	7.8

**Table 3:** Efficacy of PHMB, PHMG, PAPB, and guazatine acetate against *S. aureus*, *P. aeruginosa,* and *C. albicans* over a 24 h period formulated in contact lens base solution.

Organism	Test Compound ^a^	Average Log_10_ Reduction in Viability with Exposure (h)				
		1	2	4	6	24
*S. aureus*	PHMB	2.07	2.90	5.61 *	5.61 *	5.61 *
	PHMG	2.04	2.99	3.19	4.87	5.61 *
	PAPB	1.92	2.92	5.61 *	5.61 *	5.61 *
	Guazatine	0.01	0.01	0.35	0.58	0.72
*P. aeruginosa*	PHMB	4.27	5.63 *	5.63 *	5.63 *	5.63 *
	PHMG	2.78	5.63 *	5.63 *	5.63 *	5.63 *
	PAPB	3.16	5.63 *	5.63 *	5.63 *	5.63 *
	Guazatine	0.02	0.03	0.09	0.20	0.20
*C. albicans*	PHMB	0.68	0.69	1.13	1.41	4.05
	PHMG	0.73	1.40	3.62	5.16 *	5.16 *
	PAPB	0.16	1.22	1.50	2.07	3.90
	Guazatine	0.07	0.12	0.13	0.12	0.08

^a^ All compounds were tested at 0.05 µg/mL against *S. aureus* and *C. albicans* and 0.1 µg/mL against *P. aeruginosa*. * Demonstrates complete inactivation.

## References

[1] Cope J.R., Collier S.A., Nethercut H., Jones J.M., Yates K., Yoder J.S. (2017). Risk Behaviors for Contact Lens-Related Eye Infections Among Adults and Adolescents-United States, 2016. MMWR Morb. Mortal. Wkly. Rep..

[2] Kerr C., Chauhan K. (2022). The ACLM Contact Lens Year Book.

[3] Ung L., Bispo P.J.M., Shanbhag S.S., Gilmore M.S., Chodosh J. (2019). The persistent dilemma of microbial keratitis: Global burden, diagnosis, and antimicrobial resistance. Surv. Ophthalmol..

[4] Stapleton F., Shrestha G.S., Vijay A.K., Carnt N. (2022). Epidemiology, Microbiology, and Genetics of Contact Lens-Related and Non-Contact Lens-Related Infectious Keratitis. Eye Contact Lens.

[5] Lim C.H., Carnt N.A., Farook M., Lam J., Tan D.T., Mehta J.S., Stapleton F. (2016). Risk factors for contact lens-related microbial keratitis in Singapore. Eye.

[6] Walters R., Campolo A., Miller E., McAnally C., Gabriel M., Shannon P., Crary M. (2022). Differential Antimicrobial Efficacy of Preservative-Free Contact Lens Disinfection Systems against Common Ocular Pathogens. Microbiol. Spectr..

[7] Kilvington S., Lam A. (2013). Development of standardized methods for assessing biocidal efficacy of contact lens care solutions against Acanthamoeba trophozoites and cysts. Investig. Ophthalmol. Vis. Sci..

[8] Kovacs C.J., Lynch S.C., Rah M.J., Millard K.A., Morris T.W. (2015). Acanthamoeba encystment: Multifactorial effects of buffers, biocides, and demulcents present in contact lens care solutions. Clin. Ophthalmol..

[9] Walters R., Miller E., Campolo A., Gabriel M.M., Shannon P., McAnally C., Crary M. (2021). Differential Antimicrobial Efficacy of Multipurpose Solutions against Acanthamoeba Trophozoites. Optom. Vis. Sci. Off. Publ. Am. Acad. Optom..

[10] Papa V., van der Meulen I., Rottey S., Sallet G., Overweel J., Asero N., Minassian D.C., Dart J.K.G. (2022). Safety and tolerability of topical polyhexamethylene biguanide: A randomised clinical trial in healthy adult volunteers. Br. J. Ophthalmol..

[11] Dart J.K., Saw V.P., Kilvington S. (2009). Acanthamoeba keratitis: Diagnosis and treatment update 2009. Am. J. Ophthalmol..

[12] Heaselgrave W., Hamad A., Coles S., Hau S. (2019). In Vitro Evaluation of the Inhibitory Effect of Topical Ophthalmic Agents on Acanthamoeba Viability. Transl. Vis. Sci. Technol..

[13] Hamilton M.A., Russo R.C., Thurston R.V. (1978). Trimmed Spearman-Karber method for estimating median lethal concentrations in bioassays. Environ. Sci. Technol..

[14] Heaselgrave W., Andrew P.W., Kilvington S. (2010). Acidified nitrite enhances hydrogen peroxide disinfection of Acanthamoeba, bacteria and fungi. J. Antimicrob. Chemother..

[15] Lee J.E., Oum B.S., Choi H.Y., Yu H.S., Lee J.S. (2007). Cysticidal effect on acanthamoeba and toxicity on human keratocytes by polyhexamethylene biguanide and chlorhexidine. Cornea.

[16] Perez-Santonja J.J., Kilvington S., Hughes R., Tufail A., Matheson M., Dart J.K. (2003). Persistently culture positive acanthamoeba keratitis: In vivo resistance and in vitro sensitivity. Ophthalmology.

[17] Elder M.J., Kilvington S., Dart J.K. (1994). A clinicopathologic study of in vitro sensitivity testing and Acanthamoeba keratitis. Investig. Ophthalmol. Vis. Sci..

[18] Solel Z., Siegel M.R. (1984). Effect of the fungicides guazatine and dodine on growth and metabolism of Ustilago maydis. J. Plant Dis. Prot..

[19] Kamaruzzaman N.F., Chong S.Q.Y., Edmondson-Brown K.M., Ntow-Boahene W., Bardiau M., Good L. (2017). Bactericidal and Anti-biofilm Effects of Polyhexamethylene Biguanide in Models of Intracellular and Biofilm of Staphylococcus aureus Isolated from Bovine Mastitis. Front. Microbiol..

[20] Moore L.E., Ledder R.G., Gilbert P., McBain A.J. (2008). In vitro study of the effect of cationic biocides on bacterial population dynamics and susceptibility. Appl. Environ. Microbiol..

[21] Messick C.R., Pendland S.L., Moshirfar M., Fiscella R.G., Losnedahl K.J., Schriever C.A., Schreckenberger P.C. (1999). In-vitro activity of polyhexamethylene biguanide (PHMB) against fungal isolates associated with infective keratitis. J. Antimicrob. Chemother..

[22] Rembe J.D., Fromm-Dornieden C., Schafer N., Bohm J.K., Stuermer E.K. (2016). Comparing two polymeric biguanides: Chemical distinction, antiseptic efficacy and cytotoxicity of polyaminopropyl biguanide and polyhexamethylene biguanide. J. Med. Microbiol..

[23] Choi H., Kim K.J., Lee D.G. (2017). Antifungal activity of the cationic antimicrobial polymer-polyhexamethylene guanidine hydrochloride and its mode of action. Fungal Biol..

[24] Dreassi E., Zizzari A.T., D’Arezzo S., Visca P., Botta M. (2007). Analysis of guazatine mixture by LC and LC-MS and antimycotic activity determination of principal components. J. Pharm. Biomed. Anal..

[25] Yanai R., Ueda K., Nishida T., Toyohara M., Mori O. (2011). Effects of ionic and surfactant agents on the antimicrobial activity of polyhexamethylene biguanide. Eye Contact Lens.

